# Supplementary material for: COZOID: contact zone identifier for visual analysis of protein-protein interactions
Source: BMC Bioinformatics. 2018 Apr 6;19:125. doi: 10.1186/s12859-018-2113-6 (PMC5889581; doi:10.1186/s12859-018-2113-6)
Supplement: Supplementary file 2 — Software build. Executable binary file of the software tool. (ZIP 118,784 kb) [file 12859_2018_2113_MOESM2_ESM.zip › cozoid/java/docs/beansbinding-1.2.1-doc.zip!/org/jdesktop/beansbinding/ObjectProperty.html]

ObjectProperty (Beans Binding)


---


|  |  |  |
| --- | --- | --- |
| **Overview** \| **Package** \| **Class** \| **Use** \| **Tree** \| **Deprecated** \| **Index** \| **Help** | |  |
| **PREV CLASS**   **NEXT CLASS** | **FRAMES**    **NO FRAMES**     **All Classes** |  |
| SUMMARY: NESTED \| FIELD \| CONSTR \| METHOD | DETAIL: FIELD \| CONSTR \| METHOD |  |


---


## org.jdesktop.beansbinding Class ObjectProperty<S>

```
java.lang.Object
  org.jdesktop.beansbinding.Property<S,S>
      org.jdesktop.beansbinding.ObjectProperty<S>
```

**Type Parameters:**: `S` - the type of source object that this `Property` operates on and therefore the type of value that it represents

---

``` public final class ObjectProperty<S> extends Property<S,S> ```

An immutable, read-only, `Property` implementation whose `getValue`
method returns the source object that it is given. This class is useful when
you want to configure a `Binding` to use its source object directly,
rather than some property of the source object. For example:

```
    new SomeBindingClass(sourceObject, ObjectProperty.create(), targetObject, targetProperty);
```

Explicitly using `ObjectProperty` isn't necessary when creating `Bindings`
from this package or the `SwingBindings` package, as the set of static creation
methods include versions that handle this for you.

---

| **Method Summary** | |
| --- | --- |
| `void` | `addPropertyStateListener(S source, PropertyStateListener listener)`             Does nothing; the state of an `ObjectProperty` never changes so listeners aren't useful. |
| `static <S> ObjectProperty<S>` | `create()`             Creates an instance of `ObjectProperty`. |
| `PropertyStateListener[]` | `getPropertyStateListeners(S source)`             Returns an empty array; the state of an `ObjectProperty` never changes so listeners aren't useful. |
| `S` | `getValue(S source)`             Returns the source object passed to the method. |
| `java.lang.Class<? extends S>` | `getWriteType(S source)`             Throws `UnsupportedOperationException`; `ObjectProperty` is never writeable. |
| `boolean` | `isReadable(java.lang.Object source)`             Returns `true`; `ObjectProperty` is always readable. |
| `boolean` | `isWriteable(java.lang.Object source)`             Returns `false`; `ObjectProperty` is never writeable. |
| `void` | `removePropertyStateListener(S source, PropertyStateListener listener)`             Does nothing; the state of an `ObjectProperty` never changes so listeners aren't useful. |
| `void` | `setValue(S source, S value)`             Throws `UnsupportedOperationException`; `ObjectProperty` is never writeable. |
| `java.lang.String` | `toString()`             Returns a string representation of the `ObjectProperty`. |

**Methods inherited from class java.lang.Object**

`clone, equals, finalize, getClass, hashCode, notify, notifyAll, wait, wait, wait`

**Method Detail**

### create

```
public static <S> ObjectProperty<S> create()
```

:   Creates an instance of `ObjectProperty`.

---


### getWriteType

```
public java.lang.Class<? extends S> getWriteType(S source)
```

:   Throws `UnsupportedOperationException`; `ObjectProperty` is never writeable.

    :   **Specified by:**: `getWriteType` in class `Property<S,S>`
    :   **Parameters:**: `source` - the source object on which to operate **Returns:**: never returns; always throws `UnsupportedOperationException`; `ObjectProperty` is never writeable **Throws:**: `java.lang.UnsupportedOperationException` - always; `ObjectProperty` is never writeable **See Also:**: `isWriteable(java.lang.Object)`

---


### getValue

```
public S getValue(S source)
```

:   Returns the source object passed to the method.

    :   **Specified by:**: `getValue` in class `Property<S,S>`
    :   **Parameters:**: `source` - the source object on which to operate **Returns:**: the value of the `source` argument **See Also:**: `isReadable(java.lang.Object)`

---


### setValue

```
public void setValue(S source,
                     S value)
```

:   Throws `UnsupportedOperationException`; `ObjectProperty` is never writeable.

    :   **Specified by:**: `setValue` in class `Property<S,S>`
    :   **Parameters:**: `source` - the source object on which to operate: `value` - the new value for the `Property` **Throws:**: `java.lang.UnsupportedOperationException` - always; `ObjectProperty` is never writeable **See Also:**: `isWriteable(java.lang.Object)`, `getWriteType(S)`

---


### isReadable

```
public boolean isReadable(java.lang.Object source)
```

:   Returns `true`; `ObjectProperty` is always readable.

    :   **Specified by:**: `isReadable` in class `Property<S,S>`
    :   **Parameters:**: `source` - the source object on which to operate **Returns:**: `true`; `ObjectPropert` is always readable **See Also:**: `isWriteable(java.lang.Object)`

---


### isWriteable

```
public boolean isWriteable(java.lang.Object source)
```

:   Returns `false`; `ObjectProperty` is never writeable.

    :   **Specified by:**: `isWriteable` in class `Property<S,S>`
    :   **Parameters:**: `source` - the source object on which to operate **Returns:**: `false`; `ObjectPropert` is never writeable **See Also:**: `isReadable(java.lang.Object)`

---


### toString

```
public java.lang.String toString()
```

:   Returns a string representation of the `ObjectProperty`. This
    method is intended to be used for debugging purposes only, and
    the content and format of the returned string may vary between
    implementations. The returned string may be empty but may not
    be `null`.

    :   **Overrides:**: `toString` in class `java.lang.Object`
    :   **Returns:**: a string representation of this `ObjectProperty`

---


### addPropertyStateListener

```
public void addPropertyStateListener(S source,
                                     PropertyStateListener listener)
```

:   Does nothing; the state of an `ObjectProperty` never changes so
    listeners aren't useful.

    :   **Specified by:**: `addPropertyStateListener` in class `Property<S,S>`
    :   **Parameters:**: `source` - the source object on which to operate: `listener` - the listener to be notified

---


### removePropertyStateListener

```
public void removePropertyStateListener(S source,
                                        PropertyStateListener listener)
```

:   Does nothing; the state of an `ObjectProperty` never changes so
    listeners aren't useful.

    :   **Specified by:**: `removePropertyStateListener` in class `Property<S,S>`
    :   **Parameters:**: `source` - the source object on which to operate: `listener` - the listener to be removed **See Also:**: `addPropertyStateListener(S, org.jdesktop.beansbinding.PropertyStateListener)`

---


### getPropertyStateListeners

```
public PropertyStateListener[] getPropertyStateListeners(S source)
```

:   Returns an empty array; the state of an `ObjectProperty` never changes
    so listeners aren't useful.

    :   **Specified by:**: `getPropertyStateListeners` in class `Property<S,S>`
    :   **Parameters:**: `source` - the source object on which to operate **Returns:**: an empty array **See Also:**: `addPropertyStateListener(S, org.jdesktop.beansbinding.PropertyStateListener)`


---


|  |  |  |
| --- | --- | --- |
| **Overview** \| **Package** \| **Class** \| **Use** \| **Tree** \| **Deprecated** \| **Index** \| **Help** | |  |
| **PREV CLASS**   **NEXT CLASS** | **FRAMES**    **NO FRAMES**     **All Classes** |  |
| SUMMARY: NESTED \| FIELD \| CONSTR \| METHOD | DETAIL: FIELD \| CONSTR \| METHOD |  |


---
